# Quantitative imaging of ultrasound backscattered signals with information entropy for bone microstructure characterization

**DOI:** 10.1038/s41598-021-04425-y

**Published:** 2022-01-10

**Authors:** Chiao-Yin Wang, Sung-Yu Chu, Yu-Ching Lin, Yu-Wei Tsai, Ching-Lung Tai, Kuen-Cheh Yang, Po-Hsiang Tsui

**Affiliations:** 1 Department of Medical Imaging and Radiological Sciences, College of Medicine, Chang Gung University, Taoyüan, Taiwan; 2 Department of Medical Imaging and Intervention, Chang Gung Memorial Hospital at Linkou, Taoyüan, Taiwan; 3 Department of Medical Imaging and Intervention, Chang Gung Memorial Hospital, Keelung and Chang Gung University, Taoyüan, Taiwan; 4 Graduate Institute of Biomedical Engineering, Chang Gung University, Taoyüan, Taiwan; 5 Department of Family Medicine, College of Medicine, National Taiwan University, Taipei, Taiwan; 6 Division of Pediatric Gastroenterology, Department of Pediatrics, Chang Gung Memorial Hospital at Linkou, Taoyüan, Taiwan

**Keywords:** Ultrasonography, Biomedical engineering

## Abstract

Osteoporosis is a critical problem during aging. Ultrasound signals backscattered from bone contain information associated with microstructures. This study proposed using entropy imaging to collect the information in bone microstructures as a possible solution for ultrasound bone tissue characterization. Bone phantoms with different pounds per cubic foot (PCF) were used for ultrasound scanning by using single-element transducers of 1 (nonfocused) and 3.5 MHz (nonfocused and focused). Clinical measurements were also performed on lumbar vertebrae (L3 spinal segment) in participants with different ages (*n* = 34) and postmenopausal women with low or moderate-to-high risk of osteoporosis (*n* = 50; identified using the Osteoporosis Self-Assessment Tool for Taiwan). The signals backscattered from the bone phantoms and subjects were acquired for ultrasound entropy imaging by using sliding window processing. The independent *t*-test, one-way analysis of variance, Spearman correlation coefficient *r*_s_, and the receiver operating characteristic (ROC) curve were used for statistical analysis. The results indicated that ultrasound entropy imaging revealed changes in bone microstructures. Using the 3.5-MHz focused ultrasound, small-window entropy imaging (side length: one pulse length of the transducer) was found to have high performance and sensitivity in detecting variation among the PCFs (*r*_s_ = − 0.83; *p* < 0.05). Small-window entropy imaging also performed well in discriminating young and old participants (*p* < 0.05) and postmenopausal women with low versus moderate-to-high osteoporosis risk (the area under the ROC curve = 0.80; cut-off value = 2.65; accuracy = 86.00%; sensitivity = 71.43%; specificity = 88.37%). Ultrasound small-window entropy imaging has great potential in bone tissue characterization and osteoporosis assessment.

## Introduction

Osteoporosis is a bone metabolism disease that often occurs in old age, especially in women undergoing the menopause. When a person experiences osteoporosis, the loss of minerals in bone tissue leads to an increase in bone porosity and decrease in bone density, which in turn lead to significantly increased fracture risk^1^. The current standard for clinical diagnosis of osteoporosis is dual-energy X-ray absorptiometry, which measures bone mineral density and calculates the T score as a basis for assessing the degree of osteoporosis^2,3^. However, dual-energy X-ray absorptiometry has two major problems, namely high cost and radiation exposure. By comparison, ultrasound techniques have received attention and have also been widely used in screening for osteoporosis because of their nonionizing radiation, real-time results, portability, and cost effectiveness.

The speed of sound and slope of frequency-dependent attenuation (also called broadband ultrasound attenuation) are two acoustic parameters commonly used to characterize osteoporosis^4,5^. In practice, speed of sound and broadband ultrasound attenuation methods are typically applied to calcaneus bone measurements; they are difficult to use for evaluating central skeletal sites (e.g., the femur or spine), which are the most crucial sites of fracture due to osteoporosis^6^. In addition, the transmission of ultrasound into bone tissue results in distortion of the pulse shape, making speed of sound calculations inaccurate^7^. In comparison, cancellous bone indicates early osteoporosis^8^, and ultrasound backscatter measurement is a potential approach to cancellous bone evaluation^9–12^. Signals backscattered by cancellous bone have been shown to depend on the bone’s amount, composition, microstructure, and mechanical properties^8^. Notably, radiofrequency echographic multi spectrometry (REMS) based on the frequency-domain analysis of raw ultrasound backscattered signals acquired from a transabdominal scan of the axial sites, femur, and spine has been an emerging technique and further attracts researchers’ attention in osteoporosis assessment^13,14^. This implies that the ultrasound backscattering analysis is highly compatible with B-mode imaging for screening wide populations for osteoporosis.

Cancellous bone can be modeled as an isotropic continuum containing scatterers^15^, and random backscattered signals are formed by interactions between the incident waves and scatterers. Statistical distributions are most frequently used to describe the statistical properties of ultrasound backscattered signals for tissue characterization. Currently, a well-recognized general model of ultrasound backscattering is the homodyned K distribution^16^. The Nakagami distribution is presented as an approximation of the homodyned K distribution and has been extensively used for characterizing tissues^17^. A recent investigation used the Nakagami model to estimate the Nakagami parameter for cancellous bone characterization, indicating that the Nakagami parameter was significantly correlated with bone density and microstructure^18^. Notably, the homodyned K and Nakagami distributions were developed as backscattering models for soft tissues^17^. Evidence to support the applicability of statistical distributions derived from soft-tissue assumptions to the analysis of bone microstructures (hard tissues) is insufficient. Under this condition, a non-model-based approach that can collect information related to backscattered signals may be a more reliable and adaptive method for characterizing bones on a theoretical basis.

Among all possibilities, information entropy, a measure of signal information uncertainty or complexity, is a non-model-based method that describes changes in the backscattered statistics in a microstructure^19^. A major advantage of information entropy over conventional statistical distribution parameters is that entropy calculation can be applied to any data regardless of the data’s distribution or assumptions on the nature of the tissues to be analyzed^19,20^. Therefore, information entropy can be used as a universal method for analyzing ultrasound backscattered signals returned from soft and hard tissues. Ultrasound parametric imaging based on information entropy has been used in the quantitative analysis of soft tissues, including those of the liver^21^, eye^22^, muscle^23^, and breast^24^. The entropy-based analysis has also been applied to ultrasound characterization of backscatter from cancellous bone specimens in vitro^25^. However, the clinical feasibility of using ultrasound entropy in characterizing bone (hard tissue) and osteoporosis in vivo remains undetermined.

In this study, we assumed that ultrasound entropy imaging is useful for detecting bone’s density and microstructure. Phantom experiments and clinical measurements were performed to validate this assumption. The results revealed that ultrasound entropy imaging could be used to visualize the information uncertainty in ultrasound backscattered signals, enabling the detection of bone density and the evaluation of osteoporosis risk.

## Materials and methods

### Phantom experiments

**Figure 1:**
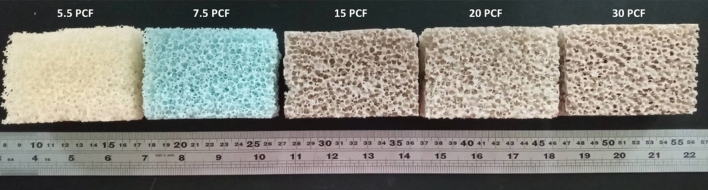
Bone phantoms composed of open-cell rigid foam. Polymer open-cell rigid foam material can be used to simulate the acoustic properties and microstructures of cancellous bone ^26^.

Five phantoms composed of polymer open-cell rigid foam (Sawbones, Vashon Island, WA, USA) were used to simulate the acoustic properties and microstructures of cancellous bone^26^. The densities of the bone phantoms were 5.5, 7.5, 15, 20, and 30 pounds per cubic foot (PCF), respectively. The properties of each phantom as specified by the manufacturer are listed in Table 1. Each phantom was cut into a material block (length: 85 mm; width: 60 mm; height: 40 mm) for ultrasound scanning and data acquisition, as shown in Fig. 1.

**Table 1:** Bone phantoms used in the study. The properties of each phantom were provided by the manufacturer.

Description	Product number	Density (g/cm^3^)	Compressive strength (MPa)	Compressive modulus (MPa)
5.5 PCF	1522-505	0.09	0.11	6.2
7.5 PCF	1522-507	0.12	0.28	18.6
15 PCF	1522-524	0.24	0.67	53.0
20 PCF	1522-526-1	0.32	1.3	105
30 PCF	1522-525	0.48	3.2	270

**Figure 2:**
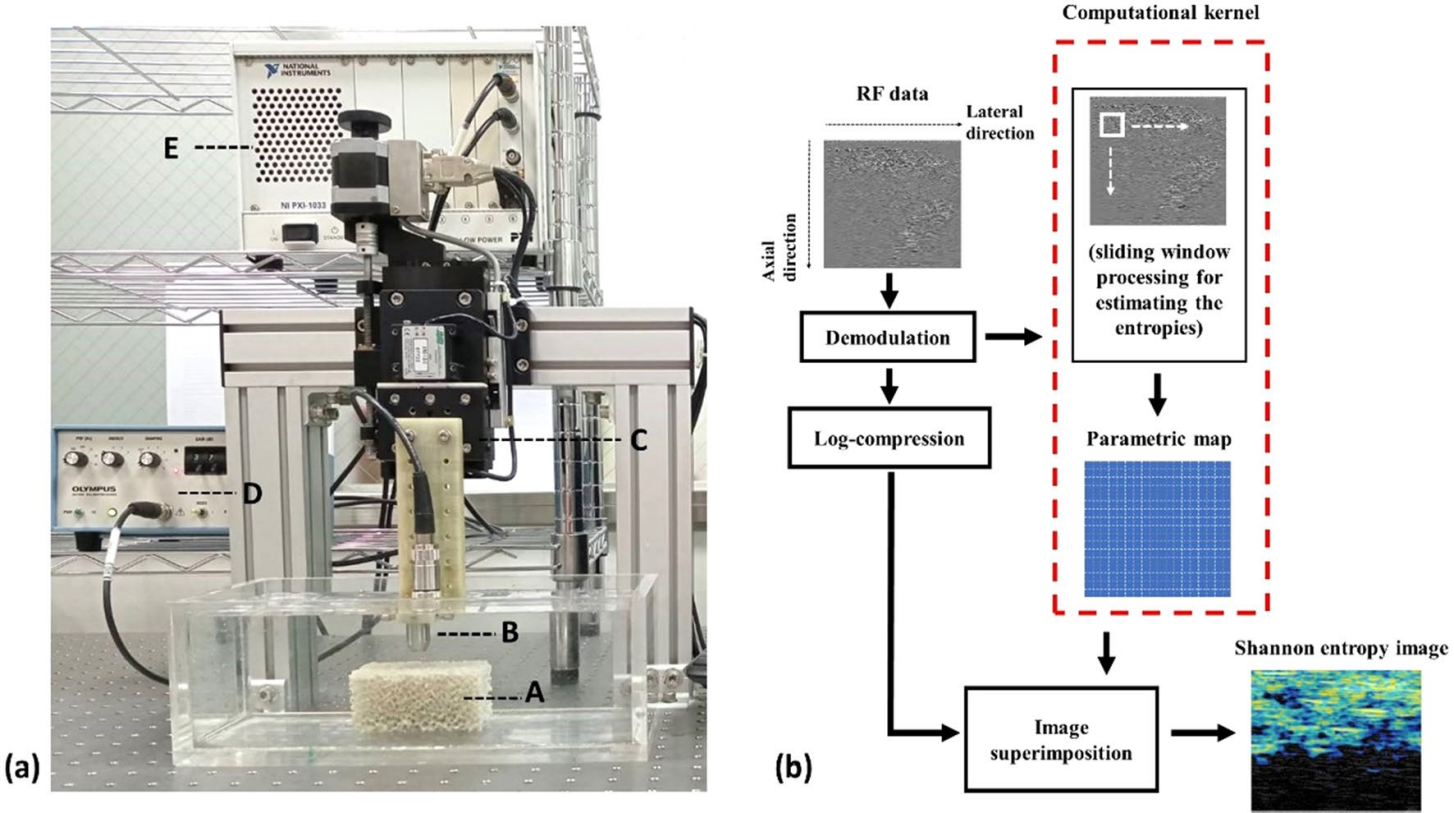
(**a**) Experimental setup for ultrasound measurements of bone phantoms, including the phantom (A), transducer (B), piezoelectric motor (C), pulser–receiver (D), and data acquisition card (E); (**b**) illustration of the algorithmic scheme of ultrasound entropy imaging by using sliding window processing.

The experimental setup for the ultrasound measurements of the bone phantoms is illustrated in Fig. 2. The ultrasound scanning system consisted of a mechanical scanning assembly, a single-element transducer, a pulser–receiver, a data acquisition card, and a computer. A high-resolution motion stage driven by piezoelectric motors (Model HR8, Nanomotion, Israel) was used to manage the scanning operation of the ultrasound transducer. The pulser–receiver (Model 5072PR, Panametrics-NDT, Waltham, MA, USA) was used to drive the transducer transmitting ultrasound, and the backscattered echoes received by the same transducer were amplified (gain: 30 dB, which was confirmed to ensure the signal amplitude values for each PCF varied within the linear range of operation and avoid the effect of signal gain saturation) and filtered (bandwidth: DC–10 MHz) using built-in amplifiers and filters in the pulser–receiver. The signals were then digitized at a sampling rate of 50 MHz by using a 8-bit data acquisition card (Model PXI-5152, National Instruments, Austin, TX, USA) for data storage on a personal computer.

Prior to measurements, the phantoms were shaken in a tank filled with water manually for one minute and then placed in the tank for one day to reduce the effects of gas bubbles (the room temperature adjusted by an air conditioner was approximately 27 °C). Each phantom was scanned using various ultrasound transducers: 1-MHz nonfocused (Model V303-SU, Panametrics-NDT), 3.5-MHz nonfocused (Model V384, Panametrics-NDT), and 3.5-MHz focused (Model V384, Panametrics-NDT) transducers. The characteristics of each transducer are listed in Table 2. The distance between the transducer and bone phantom was determined in accordance with the focal length of the 3.5-MHz focused transducer to allow the consistency of measurement distance when using the nonfocused transducers. For each phantom, 200 A-lines of backscattered radiofrequency signals were acquired to obtain the raw image data; the interval between each scan line was 100 µm. Five independent experiments were performed on each bone phantom.

**Table 2:** Transducers used in the study. The properties of each transducer were provided by the manufacturer. The bandwidth was measured by − 6 dB width of the Fourier spectrum of the incident wave obtained from the pulse-echo test. The beamwidth at focus was estimated by $2\times wavelength\times (f-number)$.

Transducer frequency (MHz)	Model no.	Focusing	Focal length (mm)	Element diameter (mm)	Beamwidth at focus (mm)	Bandwidth (MHz)
1	V303-SU	Nonfocused	N/A	12.70	6.35	0.91–1.34
3.5	V384	Nonfocused	N/A	6.35	3.18	2.26–4.28
3.5	V384	Focused	17.78	6.35	2.46	2.46–4.57

### Clinical measurements

This study was approved by the Institutional Review Boards of Chang Gung Memorial Hospital and National Taiwan University Hospital, respectively. Subjects provided informed consent, and experimental methods were performed in accordance with approved guidelines. Because age is a risk factor for osteoporosis, 34 participants were enrolled, namely Group I (age < 30 years; *n* = 20) and Group II (age ≥ 69 years; *n* = 14). Moreover, the Osteoporosis Self-Assessment Tool for Asians (OSTA) was previously proposed as a tool to assess the osteoporosis risk for postmenopausal women simply based on age and weight^27^. Therefore, 50 postmenopausal women were additionally enrolled in Group III (age > 55 years; *n* = 50) for validations of the proposed method. In this study, the Osteoporosis Self-Assessment Tool for Taiwan (OSTAi) was used as a calibrated method, and the OSTAi score was calculated as follows: $\left[age\left(\mathrm{years}\right)-weight(\mathrm{kg})\right]\times 0.2$^28^. The OSTAi scores meant the following: < − 1, low osteoporosis risk; − 1 ≤ OSTAi score < 2, moderate osteoporosis risk; and ≥ 2, high osteoporosis risk. Abdominal sagittal scanning of lumbar vertebrae (L3 spinal segment) was performed using an ultrasound imaging system (ArtUS, Telemed, Vilnius, Lithuania) equipped with a convex array transducer (C5-2H60-A5, Telemed, Vilnius, Lithuania). The transmission frequency was set to 3.5 MHz (bandwidth: 2–5 MHz), the imaging depth was 12 cm, and the focal length was 4.2 cm. Three independent scans were performed to acquire the raw image data, which consisted of 191 scan lines of radiofrequency backscattered signals (sampling rate: 40 MHz).

### Ultrasound B-mode and entropy imaging

For each raw data sample, envelope images were obtained by taking the absolute value of the Hilbert transform of the radiofrequency signals, and the corresponding B-mode images were formed using logarithm-compressed envelope images with a dynamic range of 40 dB. Concurrently, sliding window processing was applied to the raw image data for ultrasound parametric imaging on the basis of the information entropy and in accordance with the algorithmic steps described subsequently. First, a square window was set at the upper left of the image data to capture the local data points for calculating the Shannon entropy as follows^21^:

1 $${H}_{\mathrm{C}}=-\sum_{i=1}^{n}w\left({x}_{i}\right){\mathrm{log}}_{2}\left[w\left({x}_{i}\right)\right],$$

where *x*_i_ is the discrete random variable representing the backscattered data, *w*(*x*_i_) is the probability of the data value in bin *i*, and *n* is the number of bins. The window was then moved throughout the entire image in a window overlap ratio of 50% to balance image quality and computational efficiency^24^. The window side length (WSL) used for the entropy imaging was set to one to three times the pulse length (PL). The region of interest (ROI) was selected on each B-mode image and applied to the corresponding envelope and entropy images for calculating the relative amplitude (i.e., the envelope magnitude) and the average entropy, respectively. Two criteria related to the ROI selection were considered, including (i) reflection signals corresponding to the bone surfaces were avoided when selecting the ROI to ensure quantitative analysis of the internal structures in bone; (ii) the ROI falling within the bone tissue should be as large as possible to contain sufficient data (i.e., pixel values) for calculations.

### Statistical analysis

Box plots were used to display data. The normality of data was tested by the Shapiro–Wilk test. Note that the Spearman correlation coefficient identifies the strength and direction of the monotonic relationship; its use is unnecessary to consider any assumptions about the distributions of the variables^29^ and relatively robust to outliers^30^. Thus, the Spearman correlation coefficients *r*_s_ between ultrasound measurement values and PCF were calculated to confirm the monotonic relationship between the two variables. Comparisons of data between each group were performed using one-way analysis of variance (ANOVA), and those for two groups were made using the independent *t*-test. Statistical significance was considered by the probability value (*p* value) < 0.05. To evaluate the performance of ultrasound entropy in identifying the risk of osteoporosis, receiver operating characteristic (ROC) curve analysis with a 95% confidence interval was performed to obtain the area under the ROC curve (AUROC). Sensitivity, specificity, and accuracy are reported. Statistical analyses were conducted using SigmaPlot 12 (Systat Software, Inc., CA, USA).

## Results

**Figure 3:**
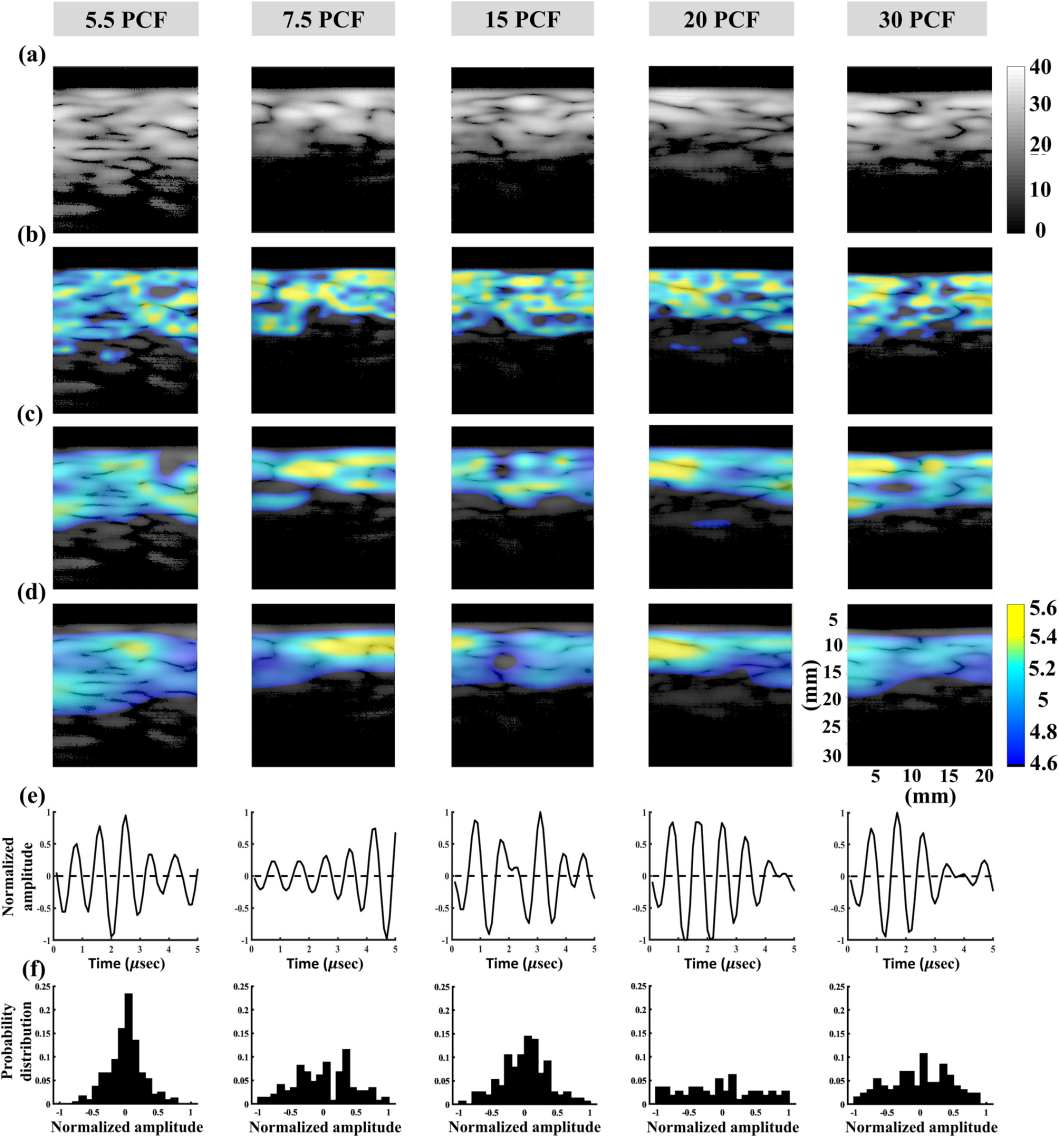
Typical 1-MHz nonfocused B-scan, entropy images, backscattered signals, and probability distributions of the bone phantoms with different PCFs. (**a**) B-mode images; (**b**) entropy images constructed using WSL = 1 PL; (**c**) entropy images constructed using WSL = 2 PL; (**d**) entropy images constructed using WSL = 3 PL; (**e**) backscattered signals; (**f**) probability distributions corresponding to signals in (**e**).

**Figure 4:**
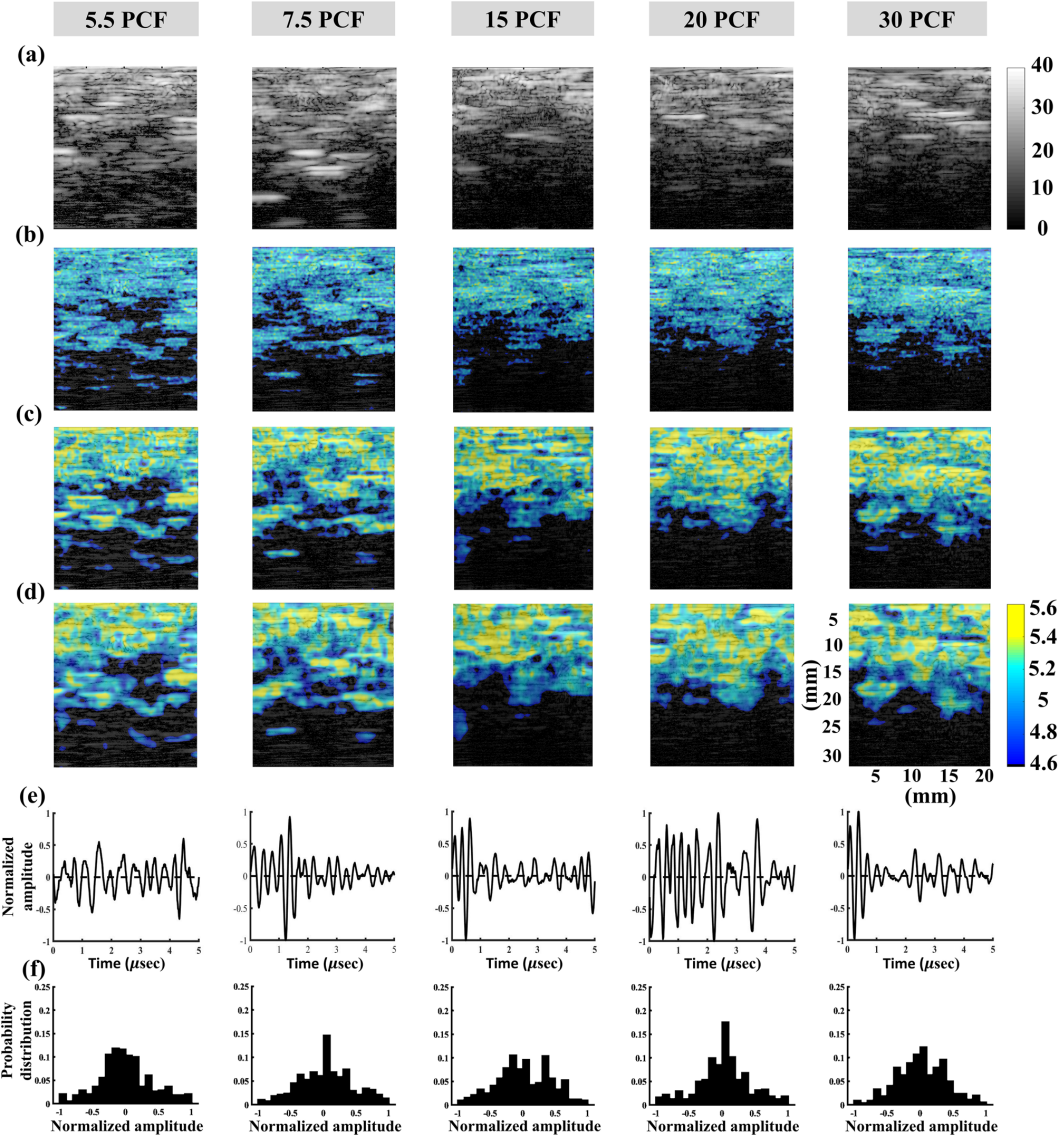
Typical 3.5-MHz nonfocused B-scan, entropy images, backscattered signals, and probability distributions of the bone phantoms with different PCFs. (**a**) B-mode images; (**b**) entropy images constructed using WSL = 1 PL; (**c**) entropy images constructed using WSL = 2 PL; (**d**) entropy images constructed using WSL = 3 PL; (**e**) backscattered signals; (**f**) probability distributions corresponding to signals in (**e**).

**Figure 5:**
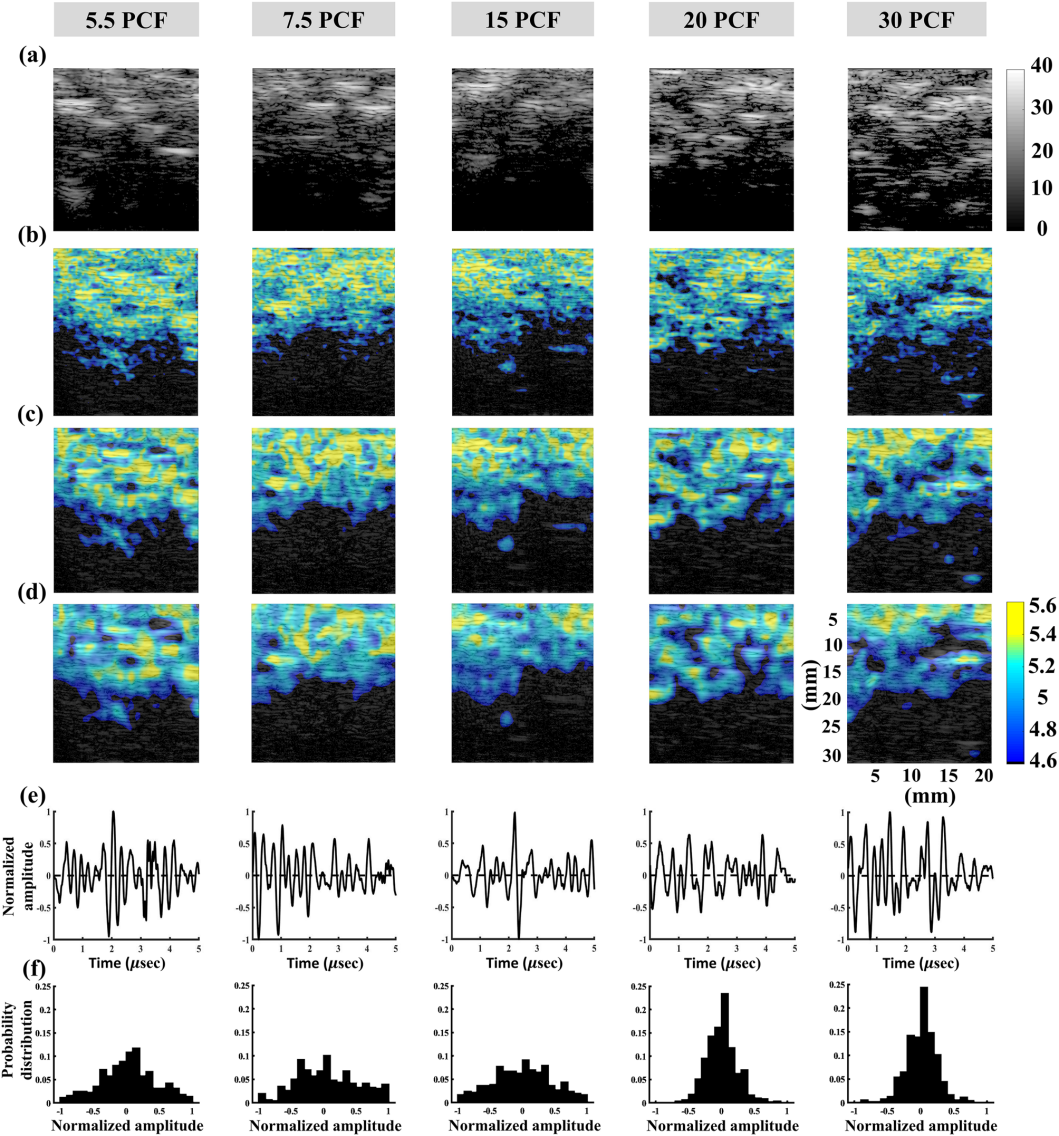
Typical 3.5-MHz focused B-scan, entropy images, backscattered signals, and probability distributions of the bone phantoms with different PCFs. (**a**) B-mode images; (**b**) entropy images constructed using WSL = 1 PL; (**c**) entropy images constructed using WSL = 2 PL; (**d**) entropy images constructed using WSL = 3 PL; (**e**) backscattered signals; (**f**) probability distributions corresponding to signals in (**e**).

Figure 3 presents typical 1-MHz B-scan, entropy images (constructed using WSL = 1, 2, and 3 PL), backscattered signals, and the probability distributions of signals obtained from the bone phantoms with different PCFs. The dependency of the brightness in the B-scan on the PCF was not significant, and those of the brightness of the entropy image as well as the signal amplitude distributions were not observed to significantly vary with the PCF. The results obtained using the 3.5-MHz nonfocused and focused transducers are presented in Figs. 4 and 5, respectively. Changes in the brightness of the B-scan were difficult to visually identify. The entropy images obtained using the 3.5-MHz nonfocused transducer behaved similarly when the PCF was increased. However, the brightness of the entropy images obtained using the 3.5-MHz focused transducer appeared to decrease when the PCF was increased from 5.5 to 30. Concurrently, the backscattered probability distribution was found to gradually decrease in the width.

**Figure 6:**
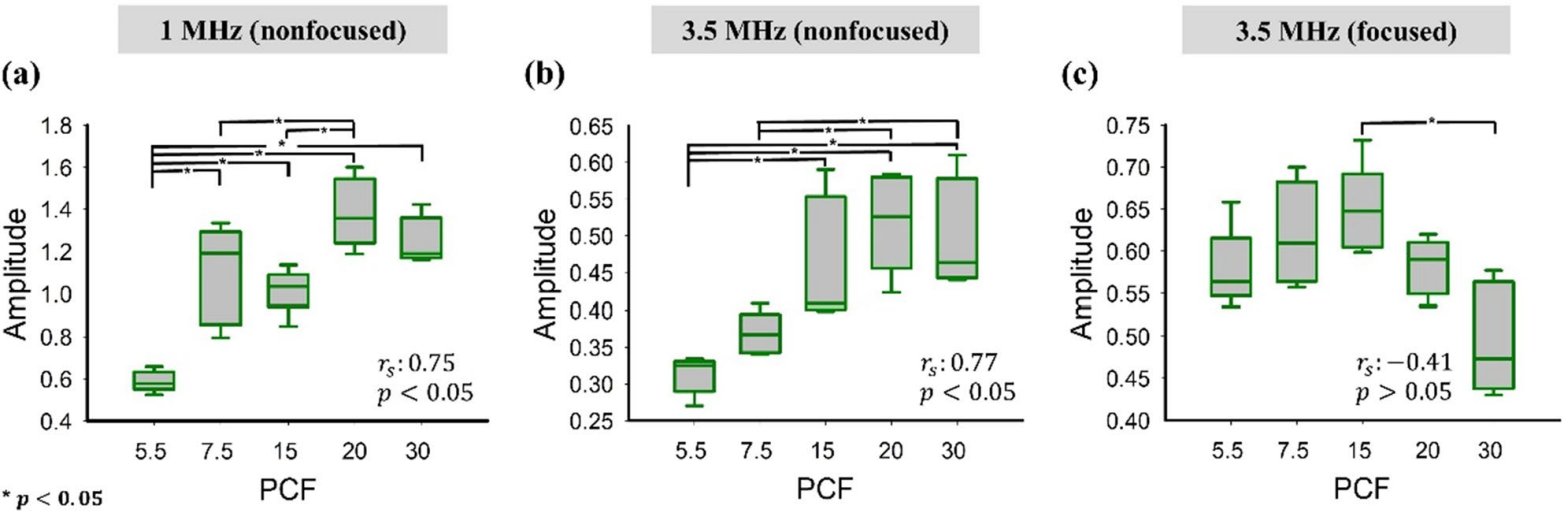
Relative amplitude of ultrasound backscattered envelope signals as a function of the PCF. The Spearman correlation analysis was performed to calculate *r*_s_ and the *p* value (a significant correlation was considered according to the *p* value < 0.05). Significant differences between data were identified by using one-way ANOVA, as indicated by the symbol ‘*’.

**Figure 7:**
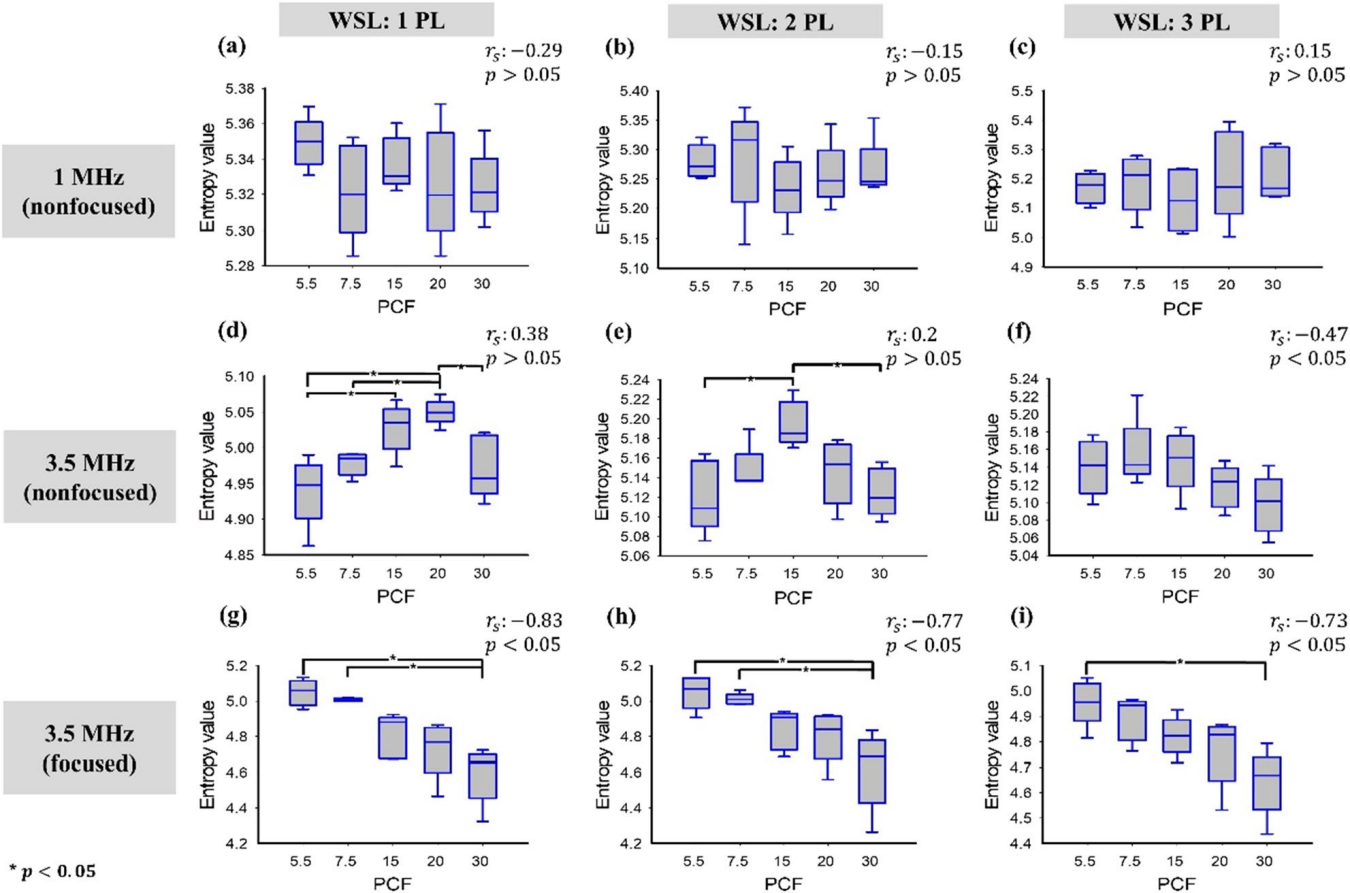
Entropy values as a function of the PCF and obtained using different transducers and WSLs for ultrasound entropy imaging. The Spearman correlation analysis was performed to calculate *r*_s_ and the *p* value (a significant correlation was considered according to the *p* value < 0.05). Significant differences between data were identified by using one-way ANOVA, as indicated by the symbol ‘*’. With the 3.5-MHz focused transducer, the entropy value monotonically decreased with increasing PCF, indicating a decrease in signal uncertainty.

The relative amplitude of ultrasound backscattered envelope signals as a function of the PCF is illustrated in Fig. 6. By increasing the PCF, the relative backscattered amplitude measured using the 1- and 3.5-MHz nonfocused transducers increased from approximately 0.6 to 1.4 (*r*_s_ = 0.75; *p* < 0.05) and from 0.3 to 0.5 (*r*_s_ = 0.77; *p* < 0.05), respectively. When using the 3.5-MHz focused transducer, the backscattered amplitude increased when the PCF was increased from 5.5 to 15 and then decreased when the PCF was greater than 15 (*r*_s_ = − 0.41; *p* > 0.05). Figure 7 displays the entropy values as a function of the PCF when using different transducers and WSLs for ultrasound entropy imaging. The entropy values measured using the 1-MHz transducer were not significantly correlated with the PCF for each WSL (*r*_s_ = − 0.29 and *p* > 0.05 for WSL = 1 PL; *r*_s_ = − 0.15 and *p* > 0.05 for WSL = 2 PL; *r*_s_ = 0.15 and *p* > 0.05 for WSL = 3 PL). The entropy was also less dependent on the PCF when the 3.5-MHz nonfocused transducer was used for entropy imaging (*r*_s_ = 0.38 and *p* > 0.05 for WSL = 1 PL; *r*_s_ = 0.2 and *p* > 0.05 for WSL = 2 PL; *r*_s_ = − 0.47 and *p* < 0.05 for WSL = 3 PL). When using the 3.5-MHz focused transducer, the entropy value monotonically decreased from approximately 5 to 4.6 when the PCF was increased from 5.5 to 30 (*r*_s_ = − 0.83 and *p* < 0.05 for WSL = 1 PL; *r*_s_ = − 0.77 and *p* < 0.05 for WSL = 2 PL; *r*_s_ = − 0.73 and *p* < 0.05 for WSL = 3 PL). These results are summarized in Table 3. Small-window entropy images constructed using the 3.5-MHz focused transducer and WSL = 1 PL resulted in an entropy value having an improved correlation with the PCF.

**Table 3:** Phantom experiment results. Data are expressed as the median and the interquartile range (^†^ normality test passed). The correlation coefficients *r*_s_ and *p* values obtained from the Spearman correlation analysis for amplitude and entropy as a function of the PCF are also provided.

Transducer	PCF	Amplitude	Entropy (WSL = 1 PL)	Entropy (WSL = 2 PL)	Entropy (WSL = 3 PL)
1 MHz nonfocused	5.5	0.58 (0.52–0.64)	5.35 (5.33–5.36)	5.27 (5.24–5.31)	5.18 (5.10–5.23)
	7.5	1.09 (0.81–1.38)	5.32 (5.28–5.35)	5.32 (5.17–5.39)	5.21 (5.06–5.30)
	15	1.03 (0.89–1.14)	5.33 (5.31–5.35)	5.23 (5.16–5.30)	5.12 (4.99–5.25)
	20	1.36 (1.18–1.58)	5.32 (5.28–5.36)	5.25 (5.19–5.32)	5.17 (5.01–5.39)
	30	1.19 (1.11–1.38)	5.32 (5.29–5.34)	5.25 (5.20–5.32)	5.17 (5.10–5.32)
		*r*_s_: 0.75 (*p* < 0.05)^†^	*r*_s_: − 0.29 (*p* > 0.05)^†^	*r*_s_: − 0.15 (*p* > 0.05)^†^	*r*_s_: 0.15 (*p* > 0.05)^†^
3.5 MHz nonfocused	5.5	0.33 (0.28–0.34)	4.95 (4.88–4.99)	5.11 (5.07–5.16)	5.14 (5.10–5.17)
	7.5	0.37 (0.33–0.40)	4.98 (4.95–4.99)	5.14 (5.11–5.17)	5.14 (5.10–5.20)
	15	0.41 (0.35–0.57)	5.03 (4.98–5.06)	5.19 (5.16–5.22)	5.15 (5.10–5.19)
	20	0.53 (0.43–0.60)	5.05 (5.02–5.07)	5.15 (5.10–5.18)	5.12 (5.08–5.14)
	30	0.46 (0.40–0.59)	4.96 (4.91–5.02)	5.12 (5.09–5.15)	5.10 (5.05–5.13)
		*r*_s_: 0.77 (*p* < 0.05)^†^	*r*_s_: 0.38 (*p* > 0.05)^†^	*r*_s_: 0.2 (*p* > 0.05)^†^	*r*_s_: − 0.47 (*p* < 0.05)^†^
3.5 MHz focused	5.5	0.56 (0.51–0.63)	5.06 (4.95–5.13)	5.07 (4.93–5.16)	4.96 (4.84–5.06)
	7.5	0.61 (0.54–0.69)	5.01 (4.99–5.02)	5.01 (4.97–5.05)	4.94 (4.78–5.00)
	15	0.65 (0.58–0.71)	4.88 (4.65–4.96)	4.91 (4.70–4.98)	4.82 (4.72–4.91)
	20	0.59 (0.54–0.62)	4.77 (4.53–4.92)	4.84 (4.62–4.98)	4.83 (4.59–4.93)
	30	0.47 (0.41–0.57)	4.66 (4.39–4.79)	4.69 (4.35–4.89)	4.67 (4.47–4.80)
		*r*_s_: − 0.41 (*p* > 0.05)	*r*_s_: − 0.83 (*p* < 0.05)	*r*_s_: − 0.77 (*p* < 0.05)	*r*_s_: − 0.73 (*p* < 0.05)

**Figure 8:**
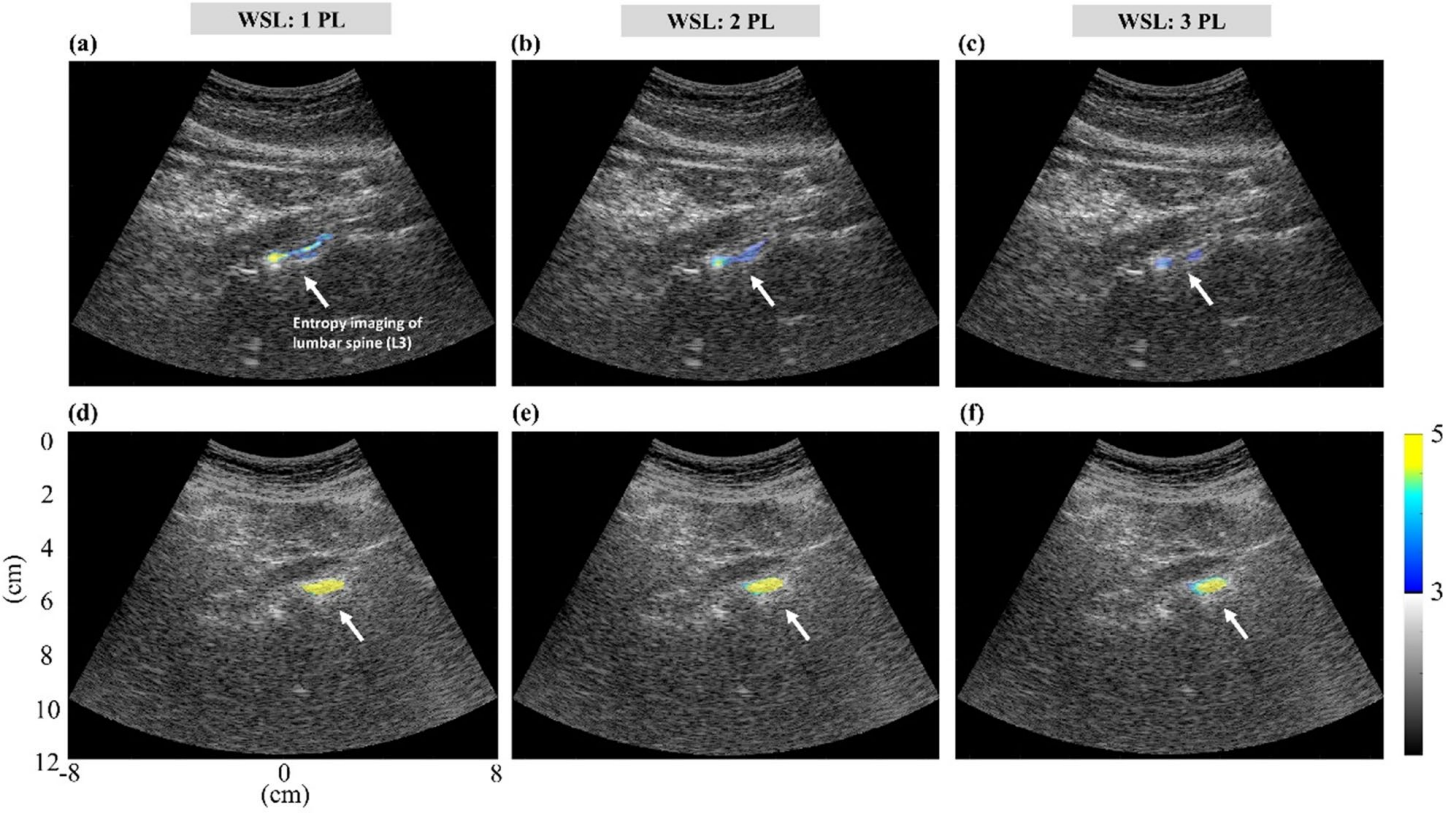
Typical ultrasound entropy images constructed using different WSLs superimposed onto the corresponding B-mode images for (**a**–**c**) Group I participant and (**d**–**f**) Group II participant.

**Figure 9:**
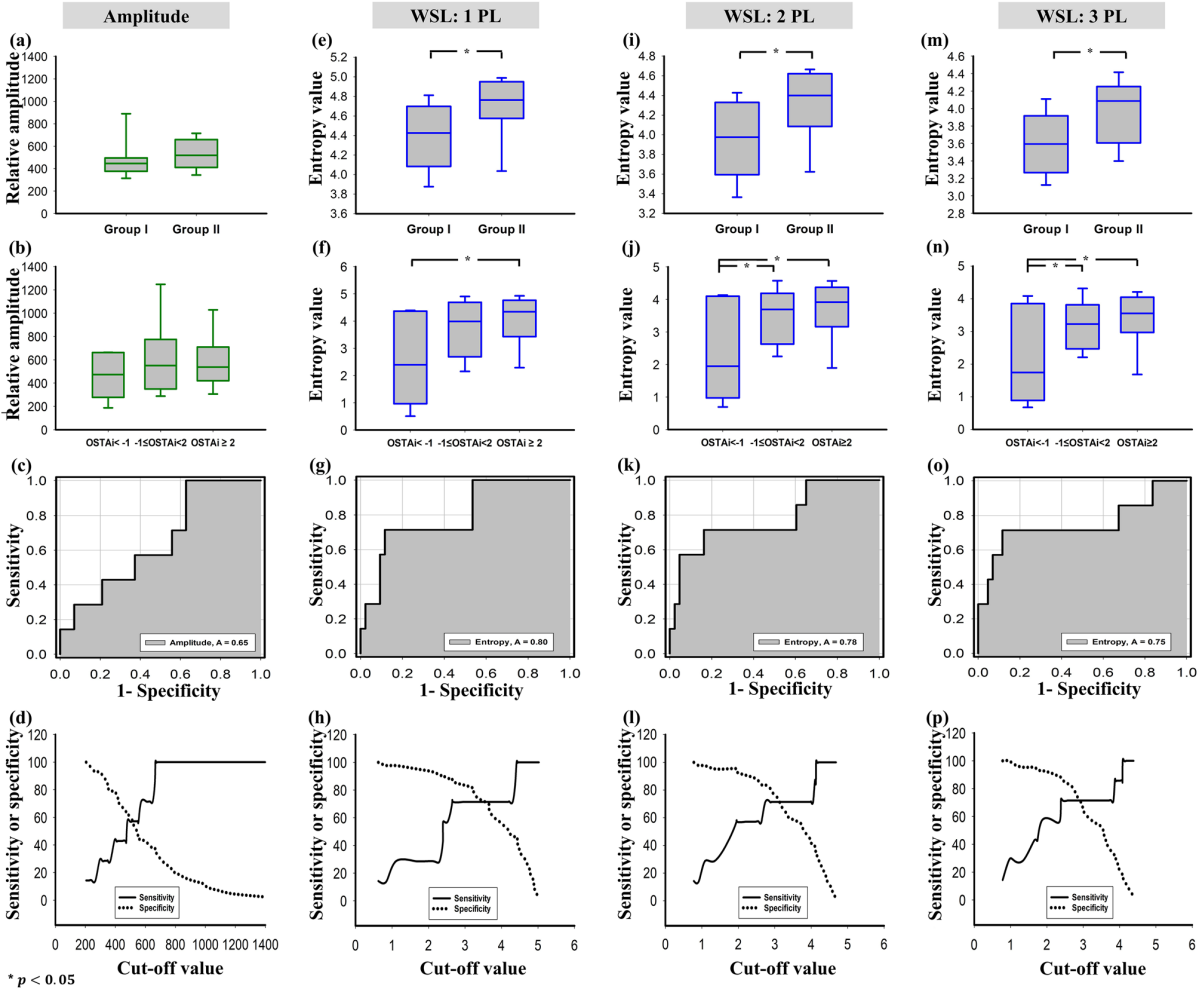
(**a**–**d**) Relative amplitude and ultrasound entropy values obtained from Groups I and II; (**e**–**h**) Relative amplitude and ultrasound entropy values obtained from subjects with different OSTAi scores in Group III; (**i**–**p**) ROC curves and sensitivity/specificity as a function of cut-off value for using amplitude and ultrasound entropy to identify moderate-to-high risk of osteoporosis (OSTAi ≥ − 1). Comparisons of data for (**a**–**d**) were made using the independent *t*-test, and those for (**e**–**h**) were performed using one-way ANOVA. The symbol ‘*’ indicates significant differences.

Table 4 displays the demographics of the participants. Figure 8 displays the images obtained from Groups I and II, respectively. For each used WSL, entropy images corresponding to lumbar vertebra L3, which were superimposed on the B-mode images, were brighter for Group II than for Group I. The difference in the B-scan and entropy image brightness between Groups I and II is illustrated in Fig. 9a–d, which indicates that the entropy value for Group II was significantly higher than that for Group I (*p* < 0.05) while the amplitude did not differ between two groups (*p* > 0.05). The postmenopausal women in Group III were regrouped in accordance with their OSTAi score for comparison, as shown in Fig. 9e–h. No significant difference in the amplitude value between low and moderate-to-high risk of osteoporosis was found (*p* > 0.05); whereas the entropy value was significantly higher for subjects at moderate-to-high risk of osteoporosis (*p* < 0.05). Refer to Fig. 9i–p. The AUROC of using ultrasound entropy imaging to identify moderate-to-high osteoporosis risk was 0.80 (cut-off value = 2.65; accuracy = 86.00%; sensitivity = 71.43%; specificity = 88.37%) for WSL = 1 PL, 0.78 (cut-off value = 2.74; accuracy = 82.00%; sensitivity = 71.43%; specificity = 83.72%) for WSL = 2 PL, and 0.75 (cut-off value = 2.39; accuracy = 86.00%; sensitivity = 71.43%; specificity = 88.37%) for WSL = 3 PL. Comparatively, the signal amplitude of the B-scan had a worse performance in characterizing osteoporosis (AUROC = 0.65; cut-off value = 666.40; accuracy = 46.00%; sensitivity = 100.00%; specificity = 37.21%). The above results represented that small-window entropy imaging improved the clinical assessment of osteoporosis risk.

**Table 4:** Patient demographics. The Osteoporosis Self-Assessment Tool for Taiwan (OSTAi) was used as a calibrated method for evaluations of osteoporosis risk.

Characteristics	Group I	Group II	Group III
No. of participants	20	14	50
**Age, years**			
Mean ± standard deviation (range)	24 ± 2.1 (21–29)	75 ± 5.6 (69–89)	65.5 ± 5.8 (55–80)
Median	24	75	66
**BMI, kg/m**^**2**^			
Mean ± standard deviation (range)	22.7 ± 3.2 (18.9–32.1)	22.4 ± 2.6 (18.6–26.7)	24.9 ± 4.5 (18.1–37.0)
Median	22.4	21.8	24.1
**OSTAi**			
Mean ± standard deviation (range)	− 8.29 ± 2.3 (− 14.8 to − 4.6)	3.81 ± 1.2 (0.98–6.4)	1.2 ± 2.6 (− 7.6 to 5.0)
Median	− 8.0	4.0	1.6

*BMI* body mass index, *OSTAi* osteoporosis self-assessment tool for Taiwan. The OSTAi score is calculated by the following formula: $\left[age\left(\mathrm{years}\right)-weight(\mathrm{kg})\right]\times 0.2$). $\mathrm{OSTAi score}\le -1$: low risk of osteoporosis; $-1<\mathrm{OSTAi score}<2$: moderate risk of osteoporosis; $\mathrm{OSTAi score}\ge 2$: high risk of osteoporosis.

## Discussion

### Study significance

Bone phantom experiments and clinical measurements for validation of the proposed method were conducted. Several milestones were reached. Ultrasound entropy was demonstrated to vary with the PCF (i.e., density) of the bone phantom and visualize change in the uncertainties of microstructure signals. The use of higher-frequency focused ultrasound (3.5 MHz) and the small-window sliding technique (WSL = 1 PL) enabled entropy imaging with a relatively strong correlation with the PCF. Through small-window entropy imaging, the subjects in Groups I and II were appropriately separated, and the risks of osteoporosis for postmenopausal women in Group III were identified with acceptable performance (AUROC: 0.80) according to a general criterion of AUROC (0.7–0.8: acceptable; 0.8–0.9: excellent; 0.9–1: outstanding)^31^. The current findings support the use of information entropy as a feasible approach for the analysis of bone tissues through ultrasound backscattering.

### Factors influencing ultrasound backscattering in cancellous bone

The mechanism associated with ultrasound backscattering from cancellous bone was extensively discussed in a review paper^32^. Cancellous bone, a meshwork of spongy tissue (trabeculae), is typically located at the core of the vertebra in the spine and at the ends of long bones (e.g., the femur or thigh bone). Architectural damage of trabecular bone caused by osteoporosis includes trabecular thinning and perforation. Acoustically, ultrasound signals backscattered from cancellous bone are contributed by the solid mineralized trabecular network and can be described by the number of scatterers and scattering cross-section per unit volume^32^, which can be treated as acoustic structural factors that characterize bone’s density and microstructure. In addition, backscattering in bone also depends on the ultrasound frequency. The ultrasound backscatter for spherical scatterers that are much smaller than the wavelength is proportional to the fourth power of the frequency and that for unresolvable cylindrical scatterers is proportional to the third power of the frequency^32^. Using higher-frequency ultrasound was suggested to improve the correlation between entropy-based analyses and bone mineral density^25^. Moreover, it has been shown that the deviation of ultrasound backscattered statistics from Rayleigh distribution depends on the variation of trabeculae diameters and the number of thin trabeculae^33^. Other factors that are able to affect backscattered statistics include porosity, the directivity of trabeculae arrangement, variations in the trabeculae thickness, and periodicity or irregularity in the arrangement of scatterers^33^.

### Physical implications and potential of entropy imaging in bone characterization

High-frequency ultrasound, transducer focusing, and small-window parametric imaging appear to be three prerequisites for effective entropy imaging that is applicable to bone microstructure characterization. Under this condition, as supported by phantom and clinical results, the relationship between the entropy and the bone density followed a monotonically decreasing function; namely, the entropy value increased with increasing the risk of osteoporosis. Some possible mechanisms are discussed below. First, osteoporosis causes an increase in porosity in the mineralized trabecular network, which may form a supportive environment for higher-frequency incident waves to interact with bone microstructures, strengthening the effect of constructive interference and increasing the uncertainty of backscattered signals. Second, the statistical nature of ultrasound backscattered signals is determined by the number of scatterers in the transducer resolution cell, which is spatially described by the pulse length and beamwidth^34^. Strong transducer focusing narrows the beamwidth and improves the spatial resolution to make changes in the number densities of scatterers relatively resolvable, enhancing sensitivity in characterizing tissues^34^. Third, parametric imaging using the conventional statistical distributions requires a large window for capturing sufficient data points to ensure stable and accurate parameter estimations. However, the accompanying boundary artifact (i.e., parameter underestimation due to the sliding window containing both strong reflection echoes from the interface and backscattered signals from the tissue parenchyma) results in low tissue characterization performance^24^. By comparison, information entropy is a relative measure of signal uncertainty, and thus the point of concern is its capability in detecting scatterer properties rather than making absolute measurements. Entropy enables the use of limited data points for estimation, supporting small-window parametric imaging to suppress boundary artifacts and improve performance^24^.

Several advantages of the proposed method can benefit bone tissue characterization and osteoporosis risk evaluation. First, lumbar vertebra segments are not large targets for image scanning. Use of the small-window technique endows parametric imaging with improved spatial resolution for characterizing the lumbar vertebra segments. Moreover, entropy imaging can be combined with abdominal standard-care ultrasound examinations to visualize the uncertainty in signals backscattered from lumbar vertebrae. The imaging of high-risk fracture sites with entropy may provide additional insights for osteoporosis assessment. Furthermore, the ultrasound entropy imaging algorithm is compatible with typical ultrasound systems, which are added in value to increase the clinical impact of developing next-generation medical systems for osteoporosis evaluation.

### Study limitations

The study has some limitations. Gas-filled pores may exist in 30-PCF phantoms, which are also higher in values of the sound speed and attenuation compared with the cancellous bone^26^. This may influence the decreasing rate for the entropy as a function of PCF but does not affect the conclusions of this study on the ability of ultrasound small-window entropy imaging on clinical evaluation of the risk in osteoporosis. In addition, the phantoms as ideal cancellous bone structures did not consider the cortical bone, which could be treated as a dense and solid material that surrounds the marrow space; such a medium mainly contributes to the production of reflection signals but is not beneficial to interact with the ultrasound wave to generate backscattered signals for tissue characterization. The image pattern corresponding to the cortical bone was difficult to recognize during clinical scanning in practice, and therefore the effect of cortical bone on estimations of the entropy value in the ROI may exist under manual ROI selection. The signal-to-noise ratio (SNR) of backscattered signals may also be influenced by the cortical bone, which absorbs the wave energy during propagation. Image segmentation techniques may be useful for identifying the sonographic features corresponding to bone tissues and may be combined with the proposed method and possible SNR enhancement strategies. Finally, the number of participants was not sufficient and without a balanced distribution, and patients with osteoporosis were not enrolled. Large-scale clinical experiments and comparisons of the proposed ultrasound entropy imaging with dual-energy X-ray absorptiometry should be performed.

## Conclusions

In this study, phantom experiments and clinical measurements were conducted to explore the feasibility of using ultrasound entropy imaging for bone tissue characterization. The results revealed that small-window entropy imaging could visualize changes in the signal uncertainty in ultrasound backscattering; the entropy value measured using 3.5-MHz focused ultrasound revealed changes in bone phantom density with higher sensitivity and performance when compared with using 1-MHz ultrasound. In clinical measurements, small-window entropy imaging of lumbar vertebrae was performed using 3.5-MHz ultrasound, and the participants with moderate-to-high risk of osteoporosis (on the basis of OSTAi score) exhibited higher entropy corresponding to higher signal complexity. Information entropy enabled small-window parametric imaging, supporting a high spatial resolution when the lumbar vertebrae were used as a target position for evaluation. Ultrasound small-window entropy imaging has great potential for future applications in osteoporosis risk evaluations. Additional clinical investigations are necessary.
